# First Autologous Cord Blood Therapy for Pediatric Ischemic Stroke and Cerebral Palsy Caused by Cephalic Molding during Birth: Individual Treatment with Mononuclear Cells

**DOI:** 10.1155/2016/1717426

**Published:** 2016-04-30

**Authors:** A. Jensen, E. Hamelmann

**Affiliations:** ^1^Campus Clinic Gynecology, Ruhr-University Bochum, Universitätsstrasse 140, 44799 Bochum, Germany; ^2^Department of Pediatrics, Ruhr-University Bochum, St. Josef-Hospital, Alexandrinenstrasse 5, 44791 Bochum, Germany; ^3^Department of Pediatrics, Evangelisches Krankenhaus Bielefeld, Kinderzentrum Bethel, Grenzweg 10, 33617 Bielefeld, Germany

## Abstract

Intracranial laceration due to traumatic birth injury is an extremely rare event affecting approximately one newborn per a population of 4.5 million. However, depending on the mode of injury, the resulting brain damage may lead to lifelong sequelae, for example, cerebral palsy for which there is no cure at present. Here we report a rare case of neonatal arterial ischemic stroke and cerebral palsy caused by fetal traumatic molding and parietal depression of the head during delivery caused by functional cephalopelvic disproportion due to a “long pelvis.” This patient was treated by autologous cord blood mononuclear cells (45.8 mL, cryopreserved, TNC 2.53 × 10*e*8) with a remarkable recovery. Active rehabilitation was provided weekly. Follow-up examinations were at 3, 18, 34, and 57 months. Generous use of neonatal head MRI in case of molding, craniofacial deformity, and a sentinel event during parturition is advocated to enhance diagnosis of neonatal brain damage as a basis for fast and potentially causative treatment modalities including autologous cord blood transplantation in a timely manner.

## 1. Introduction

Traumatic birth injury with intracranial laceration is an extremely rare event yielding a prevalence of only one newborn per a population of 4.5 million per year [1]. However, depending on the mode of injury, the resulting brain damage may lead to lifelong sequelae, for example, cerebral palsy for which there is no cure at present.

Here we report a rare case of neonatal arterial ischemic stroke after fetal traumatic head depression caused by functional cephalopelvic disproportion due to a “long pelvis” [2] during a precipitate delivery in 2004. The girl (M.H.), who was born vital with normal Apgar scores and normal umbilical arterial pH, presented a massive craniofacial deformity with depression of the left parietal bone and upper left jaw (maxilla). After six months, there were first neurologic anomalies. After 10 months right spastic hemiplegia was diagnosed and an arterial ischemic infarction with a porencephalic cyst in the central and paraventricular left white matter was confirmed by magnetic resonance imaging (MRI). Regular physiotherapy and occupational therapy were commenced on a weekly basis. At the age of five years, the patient received a transplantation of autologous mononuclear cells derived from her own umbilical cord blood that was collected at birth and stored in a blood bank. The girl recovered from hemiplegia to such an extent that she is now able to participate in endurance city runs, obtained the lifeguard certificate in swimming, rides two-wheel bicycle, and plays piano using her affected right hand.

## 2. Case Presentation

### 2.1. Medical History

A primigravid pregnant woman at term (I.H.), 33 years of age, was admitted after uneventful pregnancy to a community hospital with light irregular labor and shortening of the cervix. After preparation for delivery by enema and warm bath, rupture of the membranes occurred and hyperactive labor with strong contractions commenced. First and second stage of labor only lasted approximately 2 hours, suggesting precipitate labor. The mother gave birth to a seemingly healthy daughter with Apgar scores of 9, 10, and 10 at 1, 5, and 10 minutes, respectively, and a normal pH of 7.30 in the umbilical arterial blood. Fetal heart rate (cardiotocography, CTG) was normal during the course of labor.

The baby (M.H.) was born in a right occipitoanterior vertex presentation with head circumference of 36 cm (>97th percentile for girls [3]), length of 52 cm (>97th percentile), and birth weight of 3290 grams (50th percentile). After birth, the umbilical cord blood was collected for autologous banking. Though vital, the newborn presented a massive skull-facial deformity on the left side with depression of the parietal and maxillary bones of the left upper jaw.

### 2.2. Maternal MRI Examination of the Pelvis

**Figure 1:**
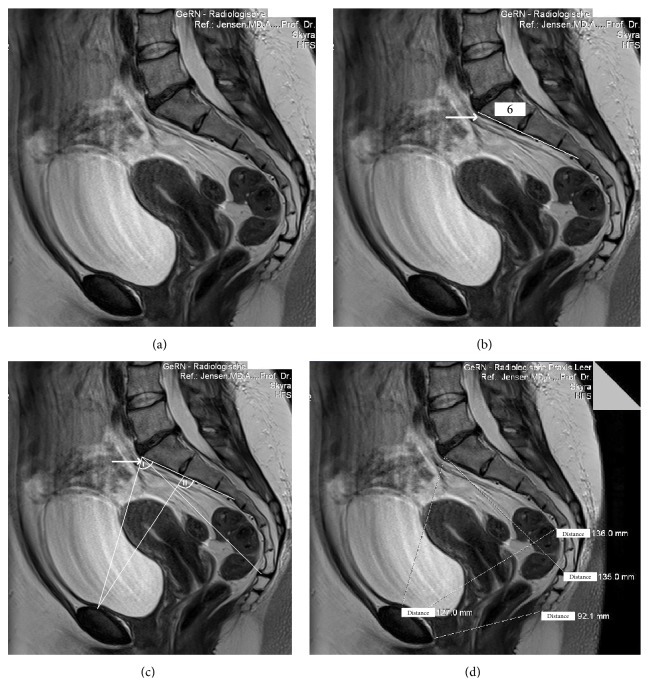
MRI of the maternal pelvis (I.H.). (a) This MRI documents a “long pelvis” [2] with assimilated (fused) lumbar vertebral body to form a sacral bone with an excessive vertebral body (arrow). (b) The “long pelvis” has 6 vertebral bones instead of the normal 5. In addition, the 6 sacral bones in the “long pelvis” fail to be concave; rather they form a straight plane (indicated by the straight line), which reduces the capacity of the mid-pelvis. (c) The pelvic inlet angle is < 90° (angle I) in a “long pelvis” [2]. (d) The distance from the top of the 6th (excessive) vertebral body to the end of the sacral bone in this “long pelvis” [2] is prolonged ((d), distance 135 mm) (3-Tesla, T1w TSE triplanar sequence).

A MRI of the maternal pelvis revealed an anomaly of the sacral bone, which normally consists of five vertebral bodies, in that the 5th lumbar vertebral body was assimilated (fused) to form a sacral bone with an excessive 6th vertebral body (Figures 1(a) and 1(b)), increasing the distance between the top of this excessive 6th vertebral body to the end of the last sacral vertebra to 13.5 cm (Figure 1(d)). This so-called “long pelvis” anomaly, named and described by Kirchhoff in 1949 [2], is rare but well known. A “long pelvis” is causing obstetrical problems in that the functional capacity of the pelvis inlet is inadequate to allow the fetus to negotiate the birth canal. Due to the high promontory, which is formed in this anomaly by the 4th lumbar vertebral body, and thus due to the steep pelvic inlet plane (angle I < 90°, i.e., the “opening angle of the pelvis” [2], Figure 1(c)), the fetal head is forced to kink the neck sideways to a greater extent than normal for head engagement (angle II > 90°, Figure 1(c)) so that the main pressure gradient primarily impacts the left parietal fetal bone in a right cephalic occipitotransverse presentation in the pelvic inlet [2]. In addition, the sacral bones in a “long pelvis” reduce the capacity of the mid-pelvis because they fail to be concave; instead, the six sacral bones form a rather straight plane, as is depicted in the MRI (Figures 1(a), 1(b), 1(c), and 1(d)). Given the large fetal head circumference of 36 cm (>97th percentile), the hampered engagement, and the strong labor that forced the head onto the pubic arch (pelvic inlet) and then through the birth canal, the depression of the soft fetal left parietal bone is comprehensible. This birth mechanism suggests excessive cephalic molding and hence a traumatic functional cephalopelvic disproportion.

### 2.3. Neurologic Examinations

The routine examinations of the newborn were normal in the first 4 months (U1–U4) except for the conspicuous faciocranial deformity. After six months, there were first neurologic anomalies (deformity of the right foot, asymmetric hand grip).

**Figure 2:**
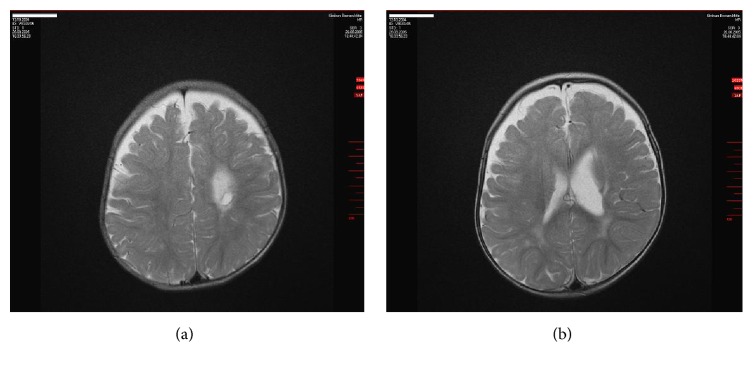
MRI of the brain (M.H.) at 10 months of age (2005). Left ischemic stroke in the central and paraventricular white matter resulting in a porencephalic cyst and enlarged lateral ventricle (1.5-Tesla Avanto, head coil array, T2w TSE sequence).

**Figure 3:**
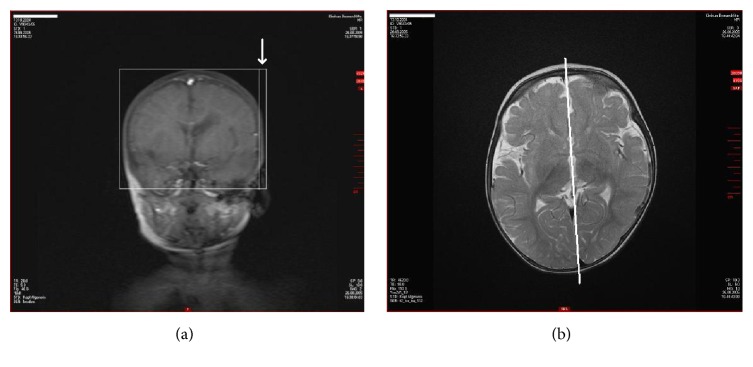
MRI of the head (M.H.) at 10 months of age (2005). (a) Significant depression of parietal bone left in a.p. projection (molding, arrow) (1.5-Tesla Avanto, head coil array, T1w TSE sequence). (b) Obvious volume reduction of the left hemisphere by birth trauma with ischemic stroke (1.5-Tesla Avanto, head coil array, T2w TSE sequence).

At 10 months of age, right spastic hemiplegic cerebral palsy was diagnosed and MRI (08/26/2005) revealed a periventricular arterial ischemic infarction with a porencephalic cyst in the left central white matter and internal capsule accompanied by an enlarged left lateral ventricle (Figures 2(a) and 2(b)). The cephalic deformity was conspicuous on MRI along with a total volume reduction of 15 mL (3.3%) in the left hemisphere as compared with the right (Figures 3(a) and 3(b)). Adding up the reduction of left hemispheric white matter, the volume of the porencephalic lesion, and the volume of the enlarged left ventricle (minus the volume of the intact right ventricle), amounted to as much as 20% (v/v), estimated total white matter loss by ischemic stroke (Figures 6(a), 6(b), 6(c), and 6(d)). The location of the lesion, affecting the left internal capsule with its long corticospinal tracts passing through, explained the obvious increase in muscular tone of the right upper and lower extremities, that is, the spasticity of the patient.

The neurologic examination confirmed spastic hemiplegia with right adductor/extensor pattern in the lower and adductor/flexor pattern in the upper extremity including pronation and ulnar deviation of the flexed wrist, flexed fingers forming a fist enclosing the adducted thumb, and the arm flexed at the elbow. The right leg was extended and adducted at the hip, slightly flexed at the knee, and plantar flexed at the ankle with inversion and external rotation of the foot. Active rolling from supine in prone position was only possible over the left side. In prone position there was supportive action of the right forearm with increased spasticity in the leg and fist closure of the fingers. Passive standing was possible, but marked equine position of the foot and increased spasticity in the right leg contributed to the difficulty in transferring weight onto the involved side and in the acquisition of standing balance. On the left side, aimed grip is swift and quick. On the right, there is delayed and slow attempted grip movement with spread fingers; in most cases object was not reached. Head circumference (MRI) was 48.0 cm (>97th percentile). The mental development was appropriate for age. In all, movement disorder with unambiguous spastic hemiplegia on the left side was noted. Intense physiotherapy and exclusion of thrombophilia were recommended. After provision of an orthosis for the right foot and ankle, free walking was possible at 15 months of age. Thrombophilia was ruled out by blood tests.

At 2.8 years of age, occupational therapy reported normal receptive and expressive speech competence and social behavior, self-confidence, curiosity, joyful playing and painting, recognition of colors, and the ability to concentrate on tasks.

Fine motor function: the left hand compensated many fine motor activities on the right, but clapping hands and waving were possible. Some supportive action of the right hand was noted. During falling, there was insufficient reflex counter-response on the right side. There was also hemineglect on the right side.

Gross motor function: she had the ability to drive Bobby car and to walk with leg orthosis and managed to walk on slopes but presented an unsteady gait. She loved slides on the playground. Overall she had improved fine and gross motor function. She intended to attend the kindergarten.

### 2.4. Autologous Cord Blood Cell Transplantation

At 4.5 years of age, the parents contacted the Campus Clinic Gynecology Bochum to inquire about a potential individual treatment with their daughter's cord blood that had been collected at birth and stored in a blood bank (Vita 34, Leipzig, Germany). After written informed consent of the parents, autologous cord blood transplantation was prepared to take place at the Department of Pediatrics, Ruhr-University Bochum, according to the German legal requirements (AMG §41(2) [BGBl.1S.2631], guideline Bundesärztekammer) as described previously [4, 5]. Identity cord blood unit/patient (CBU ID-Number 10.02.86.55.1) was confirmed genetically (Gen. number PEI G.03988.01.1).

**Figure 4:**
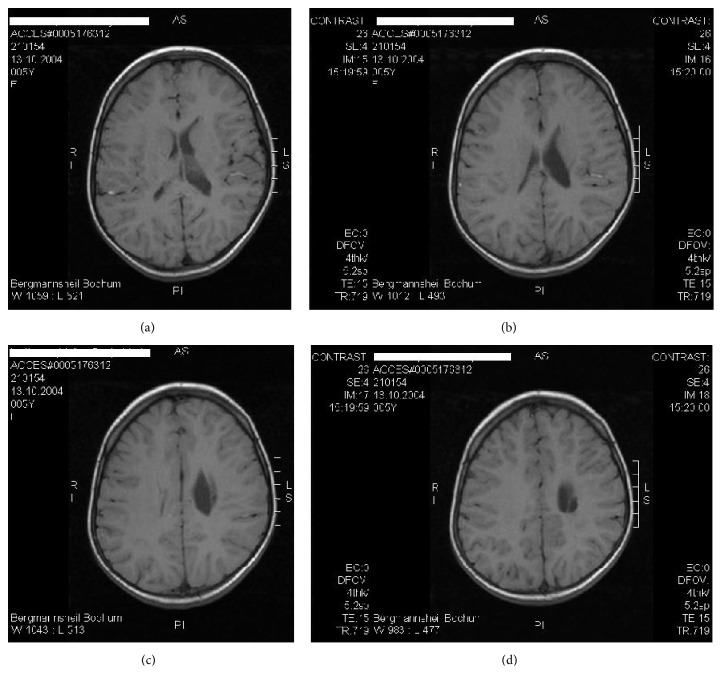
MRI of the brain (M.H.) at the age of 5 years (2009). Arterial ischemic stroke in the left central and paraventricular white matter with enlarged left ventricle before transplantation of autologous cord blood mononuclear cells (1.5-Tesla, head coil array, T1w TSE sequence).

The neurologic examination before transplantation included EEG (10:20-system) and blood tests. There was no evidence of epilepsy. The actual MRI (11/17/2009) confirmed clear evidence for an ischemic stroke with a circumscribed porencephalic cyst and minor gliosis in the left periventricular and central white matter with no evidence for residuals of cerebral hemorrhage (hemosiderin) (Figures 4(a), 4(b), 4(c), and 4(d)). The patient was shy and spoke little. There was spastic hemiplegia on the right side with increased muscle tone in the upper and lower extremities with emphasis on the upper limb. There was no monopodal jumping on the right side; only brief monopodal standing was possible. Pigeon toes on the right side internally rotated. Circumduction gait was noticed on the right hip joint. Babinski sign was positive on the right side but negative on the left side. There were no contractures, but there was slight convex scoliosis on the right side. Right arm flexed at the elbow, and pronation of the flexed wrist and slight flexion of the fingers with slight reduction of fine motor ability and strength were noticed, but segmental alternating finger movements were possible. There was hemineglect on the right side. Deep tendon reflexes (BSR, RPR, and PSR) were hyperactive on the right side, ASR was noticed on the right side also, and the same was found on the left side. Taken together, there was mild to moderate right spastic hemiplegia with comparatively minor spasticity in the lower extremity (body weight 26 kg (<97th percentile), height 123 cm (>97th percentile)).

The cord blood unit (CBU) contained 45.8 mL blood, cryopreserved by 6.0% DMSO (w/v), with a total nucleated cell count (TNC) of 2.53 × 10*e*8 mononuclear cells without erythroblasts, but including 0.72 × 10*e*6 CD34+ (15.8/mL), and 0.23 × 10*e*6 colony forming cells (CFC) (vitality 84.4%, hemoglobin 91.8 [mg/mL]). After premedication (Dimetindene Maleate 1 mg i.v., ranitidine 20 mg i.v.), the CBU was washed (Sepax S100, Biosafe, Switzerland), volume reduced, and erythrocyte depleted, and the separated unmanipulated autologous mononuclear cells (1 × 10*e*7/kg body weight) were transplanted intravenously over 15 minutes (150 mL/h). No adverse effects were noted and monitoring was continued for 24 hours. The patient was discharged and physiotherapy and occupational therapy were provided on a weekly basis. Follow-up was at 3, 18, 34, and 57 months after the transplantation of autologous mononuclear cells, respectively.

### 2.5. Outcome

Following cord blood treatment, there were some remarkable changes in motor development.

After three months, the patient presented self-consciousness and curiosity, knew all letters, wrote her name, solved easy arithmetic problems in the range one to ten, and attended a swimming course as well as the kindergarten. She used the right hand on demand to hold a spoon or a fork, but daily routine was accomplished generally with the left hand including writing. Lower leg orthosis and left shoe inlay to compensate for leg length difference (1.5–2.0 cm) were used. During slow concentrated walking, heel-to-toe movement of the centrally positioned foot was possible. Fast walking and running was also possible, but external rotation of the right arm was noted. Left heel walking was better than the right one. Walking on tiptoes on both sides was possible. Monopodal standing on the right (affected) leg was improved, though still presenting slight spastic paresis. Monopodal jumping using the right side was not possible. Slow alternating movements of the right hand were possible. Only minimal alternating finger movements on the right side were observed during high concentration. Slow pincer grip, grasping an object with all fingers, and hand opening were possible, but there was a tendency for pronation and internal rotation of the wrist. Babinski sign on the right side was positive and that on the left side was negative. The patient concentrated and was motivated during examination. Muscle tone in the right leg was only slightly increased. No significant difference in muscle strength between right and left was noted.

After 18 months, there was further intellectual and motor function improvement on the affected side. The patient solved arithmetic tasks in the range of 1 to 100. She showed good social behavior but was reluctant to share. The patient entered primary school and there was no evidence of mental retardation. She learned to ride a bicycle and there were independent walking and running with orthosis and reduced hemineglect. Monopodal standing on the right side was shortened, but standing jump was possible. Monopodal jumping on the right side was not possible. Right finger-to-nose-test and finger-to-finger-test were slow, but possible. Muscle reflexes were similar on both sides but reflex zones enlarged. The patient was pleasant, talked in full sentences, and met all requirements.

**Figure 5:**
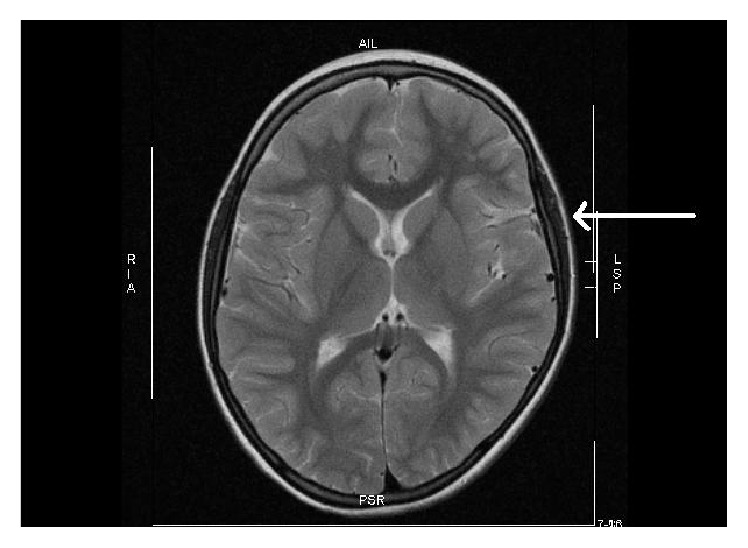
MRT of the brain (M.H.) at 7 years of age (2011). Depression of the left temporal brain after traumatic molding by a “long pelvis” [2] during parturition is still visible (arrow) (1.5-Tesla, head coil array, T2w TSE sequence).

**Figure 6:**
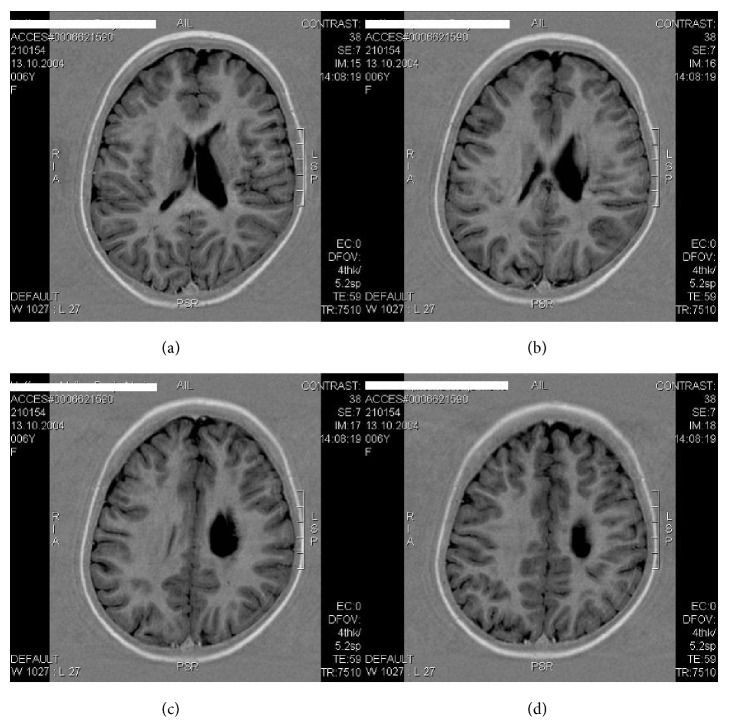
MRT of the brain (M.H.) at 7 years of age (2011). Left arterial ischemic stroke in the central and paraventricular white matter resulting in a porencephalic cyst and enlarged lateral ventricle. The estimated total white matter loss amounted to approximately 20% (1.5-Tesla, head coil array, T1w TSE sequence).

On MRI examination, 18 months after transplantation and at almost seven years of age, there was still a conspicuous depression of the patient's brain as evidenced in Figure 5 and the residuals of ischemic arterial stroke left including porencephalic cyst with only minor gliosis and enlarged left ventricle were largely unchanged as compared with the MRI before transplantation (Figures 6(a), 6(b), 6(c), and 6(d)).

After 34 months and at almost eight years of age, the patient presented further progress. There were reduced usage of orthosis, intensive swimming, frequent grasping with the right hand, and improved right muscle strength and she was binding shoelaces and having high grades at school. There was a slight residual spastic hemiplegia on the right side, insecure monopodal jumping, and monopodal standing on the right leg. Independent walking and hand usage were further improved. Though still wearing orthosis, the patient rode bicycle, learned to swim, and learned to play keyboard. Developmental psychology testing revealed an IQ score of 105, slightly above average (Hamburg-Wechsler Test, HAWIK-IV). Overall, there was tangible progress.

After 57 months, at the age of 11, and 5.5 years after autologous cord blood transplantation, the patient presented a minor neurologic residual syndrome on the right side, but now she preferred to use the right hand for daily routine with only minimally reduced fine motor control, enjoyed horseback riding, entered jogging competition without orthosis (3 km city run), earned a lifeguard certificate in swimming and diving (German Lifesaving Organisation (DLRG), gold badge), and played piano using both hands. Except for the running competitions, the patient is still wearing orthosis and awaits a transposition osteotomy to become independent of it. There is still a slightly reduced sensitivity and fine motor control of the foot and no walking on tiptoes or heels, perhaps due to the weight gain >99th percentile (63 kg, BMI 24 kg/m^2^), along with a certain weakness on the affected side.

However, rehabilitation was extraordinarily successful with activities in music, sports, full social integration among peers, and high grades at school (5th grade, secondary school). In age adjusted developmental psychology testing the patient now scored an IQ of 112 (>75 percentile, Hamburg-Wechsler Test, HAWIK-IV).

## 3. Discussion

We report a rare case of traumatic neonatal ischemic stroke with hemiplegic cerebral palsy caused by functional cephalopelvic disproportion and parietal head depression due to a “long pelvis” of the mother [2] that was treated with autologous cord blood mononuclear cells [6]. The ischemic stroke resulted from a traumatic occlusion of a penetrating thalamostriatal tributary of the left middle cerebral artery supplying the white matter beyond the basal ganglia, a unique feature of the developing brain [7]. During parturition the pressure gradient on the parietal bone and the depression were obviously sufficient to kink and/or occlude one of these small penetrating thalamostriatal arterial tributaries of the left middle cerebral artery that—unlike in the adult—provide vascular supply to the paraventricular white matter beyond the basal ganglia [7].

Due to the anatomical fact that long corticospinal nerve tracts originating from the motor cortex, which represents the whole body motor function, pass through the periventricular region and internal capsule supplied by this thalamostriatal tributary, the neurologic sequelae match the injured tracts resulting in isolated hemiplegia of the upper and lower extremities in this case.

As we have shown previously in an acute perinatal cerebral hypoxic-ischemia model in newborn rats [8, 9], mononuclear cells derived from human cord blood show a highly specific “homing” and migrate into the lesioned region of the brain in large numbers within 24 hours when given intraperitoneally [9, 10]. This chemotactic process is mediated in part by stromal-derived factor- (SDF-) 1, a chemokine that is expressed in the lesioned brain, and transplanted human umbilical cord blood cells expressing the SDF-1 receptor CXCR4 migrate to the lesioned site [11]. Thus, neurotrophic (e.g., BDNF, VEGF), synaptotrophic (e.g., NGF), anti-inflammatory (e.g., Il-6, Il-8, and Il-10), antigliotic (e.g., Cx43), antiapoptotic, and proangiogenic neuroregenerative effects are entrained resulting in significant functional neuroregeneration [12–16]. The beneficial effects of cord blood mononuclear cells observed after acute brain damage caused by hypoxic-ischemia include reduced spastic paresis and recovery of gross motor function, fine motor coordination, muscle strength, somatosensory cortical processing, and, as observed in children, recovery of cognition, vision, and active and receptive speech competence [4, 6, 9, 11, 13]. To what extent these processes may be involved in neuroregeneration after late transplantation of autologous cord blood mononuclear cells as in the present case remains to be established in further systematic clinical research.

Though causality is impossible to establish, there is clinical and experimental evidence to support the view that the patient's functional neuroregeneration observed particularly in the upper right extremity after ischemic stroke may be in part mediated by therapeutic effects of autologous cord blood mononuclear cells along with intense physiotherapy and occupational therapy. Of note is the delayed transplantation of the mononuclear cells approximately 5 years after the insult, and it may be argued that in this case the balance of evidence suggests rather a spontaneous recovery from stroke than a therapeutic effect of cell treatment. Of course, this view cannot be rebutted in an individual treatment and most of the experimental evidence votes for early intervention; however, there already exists clinical evidence for successful treatment of cerebral palsy using autologous [6, 17] and allogeneic [18] cord blood several years after the insult. The mechanisms involved in therapeutic effects after delayed mononuclear cell treatment still await elucidation. Particularly, the acute inflammatory response and chemotactic SDF-1 expression, important for the “homing” of the cells to the lesion, are likely to have ceased by that time of transplantation. Also, long-acting inflammatory responses after brain damage promoting “homing” after late cord blood transplantation have not been described as yet. However, notably, the blood-brain barrier disruption after traumatic brain injury is an early event that may persist for many years in humans [19]. This may explain in part as to why therapeutic effects are observed even after late transplantation of mononuclear cells derived from cord blood.

To our knowledge, this is the first published report on an individual autologous cord blood treatment of a pediatric ischemic arterial stroke and cerebral palsy caused by traumatic skull depression in the neonate during delivery on the basis of a “long pelvis” in the mother [2].

Interestingly, there was isolated white matter damage causing exclusive motor deficits in a child with fully developed mental, intellectual, social, and emotional capacities enabling her to excel at school performance. This is particularly noteworthy, because there was a significant hemispheric volume reduction on the affected left side by 15 mL (−3.3%) as compared to the right side, indicative of degeneration of neurones while the head circumference (MRI) at 10 months of age continued to be > 97th percentile (48 cm, [20]). Adding up the reduction in left hemispheric white matter by the lesion and the enlarged ventricle amounted to as much as 20% estimated total white matter loss by ischemic stroke. In the developing brain, it is known that hypoxic-ischemia is followed by retrograde and antegrade “Wallerian” transsynaptic degeneration of (e.g., subplate) neurones [21]. Thus, the total loss of neuronal tissue following ischemic stroke is high and renders the degree of the patient's motor recovery in the absence of mental delay in spite of late cell therapy particularly remarkable.

Hypoxic-ischemic brain lesions have recently been successfully treated in human newborns and children by autologous human cord blood mononuclear cells, demonstrating that this novel treatment modality is safe and efficacious [4–6, 17, 22, 23]. However, it seems important to combine cell treatment with intense physiotherapy and occupational therapy for maximum effect [4]. In the present case, particularly supportive for a successful rehabilitation were the absence of any mental and intellectual deficits and the high level of cognitive, language, and communication abilities. Thus, the patient's rehabilitation culminated in the ability to perform endurance runs, practicing lifeguard swimming on premier level, and playing piano using her affected hand with dexterity.

A maternal “long pelvis” is rare [2] and an isolated occlusion of a small thalamostriatal artery by depression of both the parietal skull and the infant's temporal brain is extremely rare. However, craniofacial deformation and molding of the head of newborns is a comparatively frequent observation, which is currently considered normal—a fact that merits reconsideration [24].

Given the facts that (1) 40% of the newborns presenting neonatal stroke are asymptomatic, (2) stroke is among the top ten causes of death in childhood [25–27], and (3) 50–85% of survivors of stroke will be left with long-term problems (e.g., seizures, physical disability, and speech or learning difficulties), a different management of children born with significant molding of the head and/or other sentinel events during birth seems mandatory. Generous usage of appropriate imaging techniques, particularly of head MRI, must be employed to reduce the false negative detection rate of neonatal stroke. In spite of the low prevalence of the condition, the resulting years lived with both physical and intellectual disability (YLDs) after pediatric stroke are a significant global burden [28].

Thus, neonatal cephalic molding during birth and head deformity particularly when combined with sentinel events during parturition require further examination of the head by MRI for early diagnosis of neonatal brain damage as a basis for a causative treatment of imminent cerebral palsy in a timely manner, for example, by autologous cord blood cell therapy.
